# Progressive Thinning of Visual Motion Area in Lower Limb Amputees

**DOI:** 10.3389/fnhum.2016.00079

**Published:** 2016-03-01

**Authors:** Guangyao Jiang, Chuanming Li, Jixiang Wu, Tianzi Jiang, Yi Zhang, Lu Zhao, Alan C. Evans, Lei Li, Shuhua Ran, Xuntao Yin, Jian Wang

**Affiliations:** ^1^Department of Radiology, Southwest Hospital, Third Military Medical University, Chongqing, China; ^2^Department of Rehabilitation, Southwest Hospital, Third Military Medical University, Chongqing, China; ^3^National Laboratory of Pattern Recognition, Institute of Automation, Chinese Academy of Sciences, Beijing, China; ^4^Department of Osteology, Traditional Chinese Medical Hospital of Taian, Taian, China; ^5^McConnell Brain Imaging Centre, Montreal Neurological Institute, McGill University, Montreal, QC, Canada

**Keywords:** amputation, brain plasticity, cortical thickness, V5/MT+, visual motion area

## Abstract

Accumulating evidence has indicated that amputation or deafferentation of a limb induces functional or structural reorganization in the visual areas. However, the extent of the visual areas involved after lower limb amputation remains uncertain. In this investigation, we studied 48 adult patients with unilateral lower limb amputation and 48 matched healthy controls using T1-weighted magnetic resonance imaging. Template-based regions of interest analysis was implemented to detect the changes of cortical thickness in the specific visual areas. Compared with normal controls, amputees exhibited significantly lower thickness in the V5/middle temporal (V5/MT+) visual area, as well as a trend of cortical thinning in the V3d. There was no significant difference in the other visual areas between the two groups. In addition, no significant difference of cortical thickness was found between patients with amputation at different levels. Across all amputees, correlation analyses revealed that the cortical thickness of the V5/MT+ was negatively correlated to the time since amputation. In conclusion, our findings indicate that the amputation of unilateral lower limb could induce changes in the motor-related visual cortex and provide an update on the plasticity of the human brain after limb injury.

## Introduction

Plasticity in the human brain can occur rapidly as a consequence of peripheral lesions or sensory deprivation, such as amputation (Cohen et al., 1991; Schwenkreis et al., 2001). Previous studies largely focused on plasticity in the primary sensorimotor cortex of the hemisphere contralateral to the amputation (Flor et al., 2001; Xie et al., 2013), which has been shown to correlate with phantom limb pain (PLP) and been considered to reflect maladaptive plasticity (Maciver et al., 2008; Houze et al., 2013). On the other hand, mirror training could alleviate PLP, suggesting that pain and cortical reorganization can potentially be altered by visual feedback (Brodie et al., 2003; Moseley, 2005; Chan et al., 2007). In addition, unilateral arm amputees show impaired spatial perception near their affected hand (Makin et al., 2010), as well reduction in visuomotor object affordances (Wilf et al., 2013). Therefore, plasticity following limb amputation is not only restricted to local remapping within the sensorimotor region but also extends to the brain regions associated with visual information processing.

Our understanding of the functional and structural organization of visual information processing has evolved considerably in the last decade. Initially, anatomical and lesion studies revealed that outputs from the primary and secondary visual cortex (V1 and V2) to middle temporal complex (V5/MT+) and visual area 4 (V4) initiate two segregated but interacting parallel processing streams, i.e., the dorsal “where/how” and ventral “what” visual streams. A rough summary of their function is that the ventral stream represents vision for perception, while the dorsal stream represents vision in service of action (Wandell et al., 2007). The dorsal stream may actually consist of two relatively segregated subcircuits (Saur et al., 2008). The dorso–dorsal pathway provides the dominant input to the superior parietal lobule through visual area 6 (V6) and plays an important role in goal-directed visuomotor transformation, such as reaching and grasping, whereas the ventral–dorsal pathway through V5/MT+ to the inferior parietal lobule is assumed to be relatively more involved in multimodal motion detection and tool use (Binkofski and Buxbaum, 2013; Pitzalis et al., 2013; Van Kemenade et al., 2014; Yu et al., 2014; Ajina et al., 2015). Newer findings emphasize the role of area V3ab in motion processing and its role in the dorsal stream (Serences and Boynton, 2007).

Although few studies have reported that amputees presented gray matter changes in the visual areas, it is still unclear which subregions are specifically involved. Besides, most of existing studies included only patients with upper limb amputation, and the brain reorganization following lower limb amputation might be distinguishable as the functions and brain circuits of the upper and lower extremities are differentiated. We hypothesized that amputation at the lower limb can result in neural changes in the specific visual areas that are measurable with brain imaging. In order to systematically characterize the brain reorganization, the mean cortical thickness in each of the visual areas was measured using surface-based morphology and regions of interest (ROI) approach. In addition, the relationships between imaging measures and clinical variables were also investigated.

## Materials and Methods

### Participants

Forty-eight adult patients (38 males and 10 females) with unilateral lower limb amputation were recruited consecutively and prospectively from the Prosthetic and Orthotic Clinics at the Department of Rehabilitation, Southwest Hospital in Chongqing between December 2012 and June 2015. Thirty-two patients were amputated following traumatic injury and the others were due to tumors or osteomyelitis (two melanoma, five osteosarcoma, one ameloblastomas, and eight osteomyelitis). All the patients had been fitted with prostheses. The PLP and stump pain was assessed by the five-category verbal rating scale (Lund et al., 2005). Exclusion criteria were: (1) age at amputation or magnetic resonance imaging (MRI) of less than 18 years or more than 60 years; (2) amputation at another part of the body; (3) history of brain injury due to trauma; (4) presence of major systemic disease (e.g., diabetes mellitus or cardiovascular diseases), psychiatric, or neurological illnesses; (5) duration between amputation and MR scanning of less than 1 month.

Forty-eight age- and sex-matched healthy controls without neurological or psychiatric diseases and with normal brain MRI were recruited from the local community. All the participants were dominantly right-handed as determined by the Edinburgh Handness Inventory (Oldfield, 1971) and had a score of 27 or higher on the Chinese version of the Mini-Mental Status Examination (MMSE) (Zhang et al., 1990). The study was approved by the Medical Research Ethics Committee of Southwest Hospital, and written informed consent was obtained from all participants.

### Structural MRI Acquisition

The MRI experiment was performed using a 3-Tesla scanner (Magnetom Trio, Siemens, Erlangen, Germany) with an 12-channel phased-array head coil. The subjects were required to close their eyes and avoid any movement during the image acquisition. 3D high-resolution structural images were obtained using a T1-weighted magnetization prepared rapid acquisition gradient echo (MPRAGE) sequence (repetition time = 1,900 ms, echo time = 2.52 ms, inversion time = 900 ms, flip angle = 9°, matrix = 256 × 256, thickness = 1.0 mm, 176 slices with voxel size = 1 mm × 1 mm × 1 mm).

### MRI Data Processing

Cortical reconstruction and volumetric segmentation was performed with FreeSurfer (version 5.3.0, http://surfer.nmr.mgh.harvard.edu). The automated processing stream mainly included removal of non-brain tissue (Segonne et al., 2004), Talairach transformation, segmentation of gray/white matter tissue (Fischl et al., 2004), intensity normalization, topological correction of the cortical surface (Saur et al., 2008), and surface deformation to optimally place the tissue borders (Fischl and Dale, 2000).

**Figure 1:**
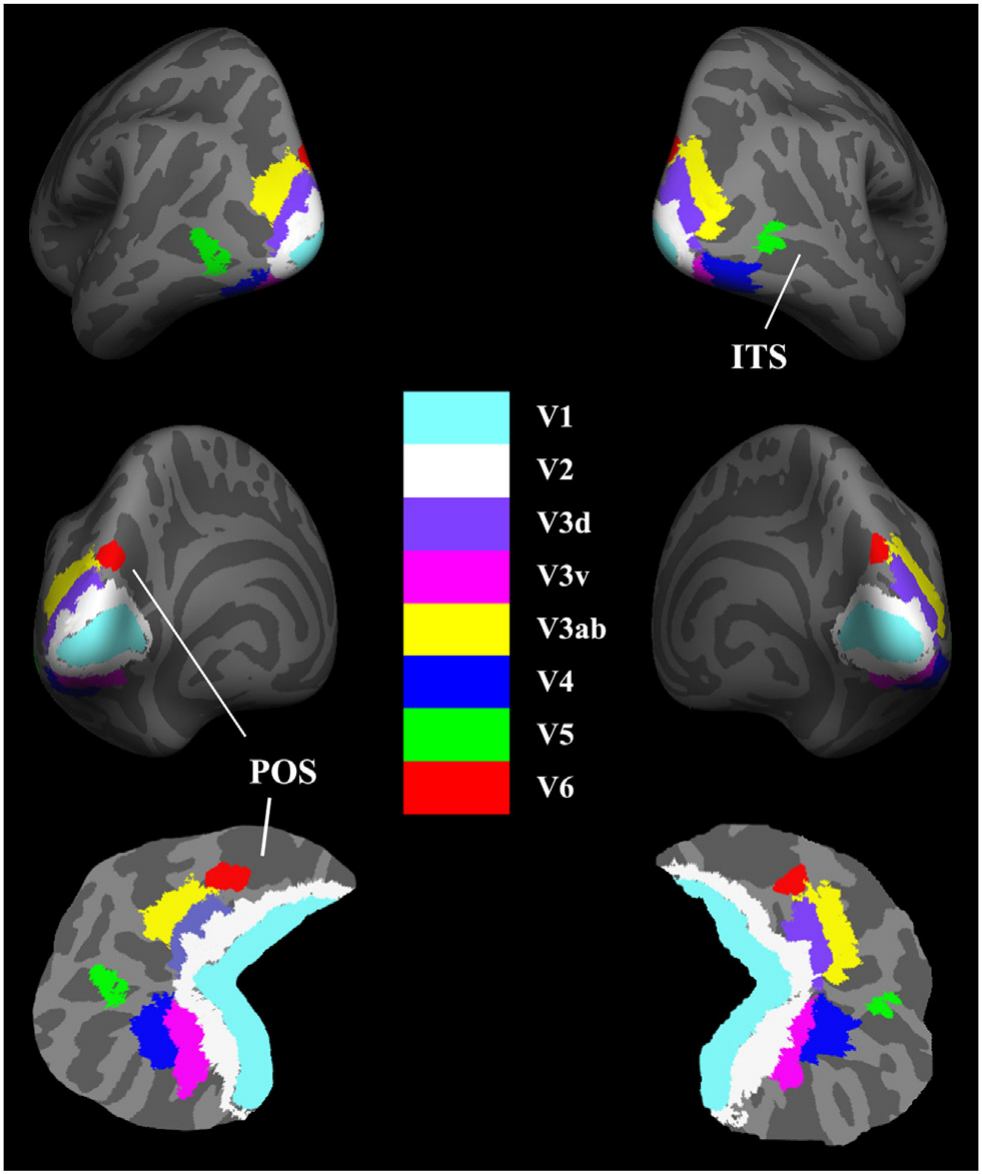
**Maps of visual areas shown in lateral, medial, and flattened views of the bilateral hemispheres (RH)**. Light gray indicates gyri (convex curvature); dark gray indicates sulci (concave curvature). The area V6 adjoins the borders of the medial-most parts of V3d and V3ab. ITS, inferior temporal sulcus; POS, parietal–occipital sulcus.

After creation of the cortical representations, the visual areas were parcellated based on existing atlases. The delineation of the V1 label was based on the study by Hinds et al. (2008) and corresponded to Brodmann area (BA) 17, and the V2 label was described by Fischl et al. (2008) and corresponded to BA 18. The delineation of the V5/MT+ label was based on the work of Malikovic et al. (2007). This V5/MT+ area was located close to the intersection of the anterior occipital and the inferior lateral occipital sulci in the region of the temporo-occipital junction. To avoid the overlap among these labels, the above atlases were all thresholded at 80% probability. As the V3, V4, and V6 templates are not available in FreeSurfer, we extracted the V3ab, dorsal V3 (V3d), ventral V3 (V3v), and V4 using the probabilistic atlas of retinotopic visual areas, which was constructed based on the object mapping data from 15 subjects (Henriksson et al., 2012). The human V3ab borders the anterior portion of V3d. The V3v is buried deep in the collateral sulcus, and V4 is located on the lateral bank of this sulcus but also reaches the fusiform gyrus in the occipital section. The V6 was described by Pitzalis et al. (2006) and located on the dorsal margin of the parieto-occipital sulcus. The labels were registered to the surface of each subject using the spherical parameters of the cortical surface (Fischl et al., 1999). For each subject, the eight visual areas were visually inspected for any inaccuracies in parcelation and manually corrected if necessary. The final parcelation of these visual cortices was illustrated in Figure 1.

Cortical thickness was calculated as the average of the distance from the white matter surface to the closest point on the pial surface and from that point back to the closest point on the white matter surface (Fischl and Dale, 2000). The thickness maps produced are not limited to the voxel resolution of the image due to the interpolation procedure (Fischl and Dale, 2000) and thus sensitive to sub-millimeter differences between groups. For each visual area, we computed the average cortical thickness across the unilateral area.

### Statistical Analysis

Continuous variables were tested for normality using the Shapiro–Wilk test. Differences in the demographic measurements were assessed using two sample *t*-tests, and the chi-square test was used for gender. Exploratory two sample *t*-tests were conducted to investigate the possible differences of cortical thickness for each of visual areas between patients with amputation at the left side and those at the right side, and no significant difference was found. Therefore, we computed the mean cortical thickness values for the homogenous regions across the two hemispheres. Then, multiple analyses of covariance (MANCOVAs) were conducted to examine the differences of the cortical thickness measurements between amputees and normal controls, using age and gender as covariates. The results were corrected for multiple comparisons using false discovery rate (FDR) correction. A two-tailed *p* < 0.05 was considered statistically significant.

Furthermore, mixed-model multivariate analysis of variance (MANOVA) designs with amputation side (left vs. right) and amputation site (femur vs. tibia) as between-group factors, age and gender as covariates, and the different subregions of the visual cortex as repeated measures were used to investigate the effects of confounding variables on the imaging measurements in amputees. Finally, spearman correlation analyses adjusted for age and gender were used to explore the associations between the clinical variables and the mean cortical thickness of bilaterally homologous visual areas across the whole patient group. All of the statistical analyses were conducted using SPSS software (version 18.0, Chicago, IL, USA).

## Results

### Demographic and Clinical Data

The demographic characteristics were summarized in Table 1. There was no significant difference in age, gender, education level, and MMSE score between the amputees and normal controls. Twenty-two patients suffered amputation at the left side and 26 were amputated at the right. Twenty-three amputations occurred at the transfemoral and 25 at transtibial levels. PLP was present in 14 patients and 10 patients were suffering stump pain.

**Table 1:** **Demographic data of the participants**.

Characteristics	Amputees (*n* = 40)	Controls (*n* = 40)	*p* value
Gender (male/female)	38/10	36/12	0.81
Age (years)	40.0 ± 13.0 (range: 18–60)	39.9 ± 12.3 (range: 18–60)	0.96
Education (years)	9.2 ± 4.0	9.5 ± 3.4	0.69
MMSE score	28.2 ± 1.4	28.4 ± 1.3	0.47
Time since amputation (months)	57.3 ± 81.8 (range: 1–336)	–	–
Amputation at left/right	22/26		

*The data were presented as mean ± SD. MMSE, Mini-Mental Status Examination*.

### Cortical Thickness Differences

The TIV (controls vs. amputees: 1.54 ± 0.14 vs. 1.51 ± 0.11 L, *p* = 0.21) or total gray matter volume (0.65 ± 0.06 vs. 0.64 ± 0.05 L, *p* = 0.41) of amputees did not differ from that of controls significantly. Differences in cortical thickness between the two groups for each visual region are shown in Table 2. Compared with normal controls, amputees had significantly lower thickness in the V5/MT+ (*p* = 0.03) (Table 2). The V3d also exhibited a thinning trend (*p* = 0.055) in patients. There was no significant difference in the other visual areas between the two groups. Further MANOVA model found no significant effects of amputation sides (*p* = 0.76), sites (*p* = 0.12) or their interaction (*p* = 0.49) on the cortical thickness.

**Table 2:** **Comparison of cortical thickness in the visual regions**.

Regions	Cortical thickness (mm)		*p* value
	Amputees	Controls	
V1	1.66 ± 0.10	1.66 ± 0.10	0.83
V2	1.89 ± 0.11	1.91 ± 0.09	0.41
V3d	1.99 ± 0.15	2.05 ± 0.15	0.052
V3v	2.08 ± 0.16	2.09 ± 0.13	0.57
V3ab	2.10 ± 0.12	2.12 ± 0.14	0.75
V4	2.49 ± 0.14	2.49 ± 0.18	0.87
V5	2.36 ± 0.20	2.44 ± 0.16	**0.03**
V6	2.09 ± 0.17	2.09 ± 0.19	0.96

*Bold indicates *p* ≤ 0.05. The cortical thickness was presented as the mean value between the two hemispheres*.

### Correlations between Cortical Thickness and Clinical Measurements

**Figure 2:**
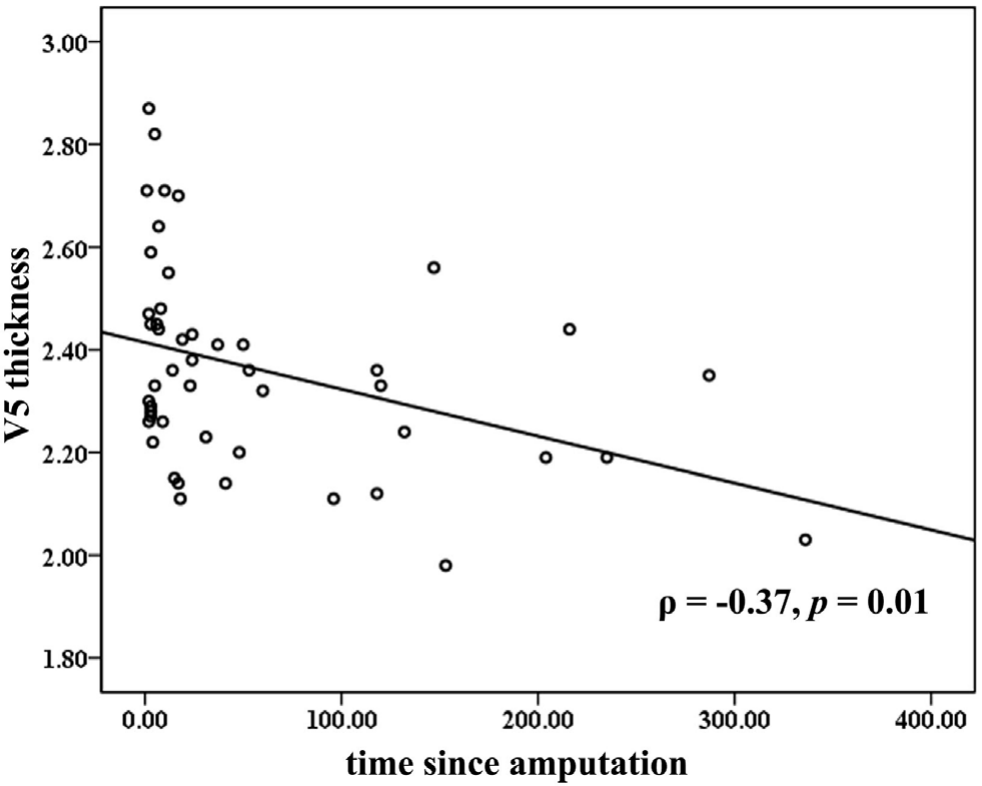
**Correlations between mean cortical thickness of the bilateral V5 and time since amputation**. The partial correlation coefficient (ρ) was corrected for age and gender.

Across all the amputees, partial correlation analyses revealed that the cortical thickness of the V5/MT+ was negatively correlated to the time since amputation (ρ = −0.37, *p* = 0.01; Figure 2). No significant correlation was found between the cortical thickness of the visual areas and the levels of PLP or stump pain.

## Discussion

In the present study, we explored brain structural reorganization in lower limb amputees. Cortical thickness was used as the proxy to evaluate the gray matter structural changes in visual areas in amputees compared with normal controls. We found that patients with lower limb amputation exhibited reduced cortical thickness in the V5/MT+ area. Additionally, correlation analyses revealed that the cortical thickness of the V5/MT+ was negatively correlated to the time since amputation, indicating that the visual cortex exhibited progressive thinning after lower limb amputation.

V5/MT+ projects to a number of satellite areas including the fundus of the superior temporal area and multiple parts of the medial superior temporal area (Borroni et al., 2008; Kolster et al., 2010; Becker et al., 2013). Studies on the electrophysiological properties of neurons in the V5/MT+ showed that a large portion of cells are tuned to the speed and direction of moving visual stimuli (Maunsell and Van Essen, 1983; Bridge et al., 2014). Functional characteristics of the V5/MT+ cluster also include responsiveness to moving visual stimuli (Kamitani and Tong, 2006; Van Kemenade et al., 2014). These results suggested that neurons in V5/MT+ play a significant role in the motion perception, and enable subjects to perceive where objects are located in the peripheral space. On the other hand, V5/MT+ also contains direction-specific information about tactile moving stimuli (Van Kemenade et al., 2014). Furthermore, V5/MT+ overlaps considerably with the extrastriate body area (EBA) in the posterior inferior temporal sulcus and the middle temporal gyrus, which are recruited in body detection and perception of body movement (often referred to as “biological motion”) (Peelen and Downing, 2005; Weiner and Grill-Spector, 2013; Palermo et al., 2014).

Human V5/MT+ is especially sensitive to the changes of the individual health or experience. Previous imaging studies revealed increased gray matter density in the visual motion areas in expert jugglers (Draganski et al., 2004; Gerber et al., 2014), or after training of processing speed (Takeuchi et al., 2011). The positive neuroanatomical plasticity in the V5/MT+ might be associated with enhanced demand for visual motion perception or eye-hand coordination. In contrast, the cortical atrophy of the V5/MT+ area in lower limb amputees might be related to the blockage of motion perception, especially for the tactile moving modality, or the absence of body detection and eye-limb coordination due to the loss of limb. Biological interpretation of the imaging changes might be attributed to the synaptic and dendritic changes, and/or the decrease in local vasculature (Scholz et al., 2009).

Negative association between the time since amputation and cortical thickness of the V5/MT+ was also detected in the lower limb amputees. Some recent MRI morphologic studies have demonstrated brain reorganization in either thalamus (Draganski et al., 2006; Makin et al., 2015) or visual streams (Preissler et al., 2013) following amputation. However, the majority of these studies mainly focused on the patients with upper limb amputation, and did not found the progressive changes occurring in the corresponding regions. As there was no significant difference in the V5/MT+ thickness in patients with amputation at the transfemoral and transtibial levels, the atrophy may not be related to the loss of knee coordination. Future rehabilitation treatments should pay attention to the progressive changes in V5/MT+ area, and validate whether the atrophy could be reversed/modulated by motion exercises or prosthesis use. Besides, no significant difference was observed in the visual areas between the right and left lower limb amputees. On possible explanation is that the visual cortices of the human brain exist interaction between hemispheres (Stephan et al., 2007). Previous studies also found substantial connectivity between the bilateral V5/MT+ (Ninomiya et al., 2011), and unilateral V1 damage would result in the alternation of cortico-cortical connection between V5/MT+ bilaterally (Bridge et al., 2008).

Parallel visual pathways in the primate brain, known as the dorsal and ventral streams, receive retinal inputs mainly through the magnocellular and parvocellular layers of the lateral geniculate nucleus. Growing evidence suggests that the functional specialization of the two cortical visual pathways may not be as distinct as it was proposed originally. Both ventral and dorsal visual streams contribute to shape perception, but that location processing appears to be essentially a function of the dorsal visual pathway (Zachariou et al., 2014). The negative findings of the reorganization in the ventral visual stream suggested that the ability of form perception was not disrupted in lower limb amputees.

Rizzolatti and Matelli (2003) proposed that the dorsal stream may actually consist of two relatively segregated subcircuits: the dorso–dorsal pathway through V6 and the superior parietal lobule concerned with the control of action “online” and the ­ventral–dorsal pathway through V5/MT+ and the inferior parietal lobule mediated space perception and “action understanding.” Neuroimaging studies on patients with optic ataxia, as characterized by the deficits in online motor control, such as reaching and grasping, highlighted the specificity of the superior parietal region and the parieto-occipital junction for direct goal-directed visuomotor transformations (Binkofski and Buxbhaum, 2013). Therefore, previous findings on the gray matter increase in these areas in upper limb amputees (Preissler et al., 2013) could be due to the compensatory adaptation for online motor control. In contrast, the reaching and grasping performance should by definition be preserved in lower limb amputees, and thus we did not found the abnormal cortical changes in the dorso-dorsal stream.

### Limitations and Future Directions

There were some limitations in our study. First, only a small number of patients were recruited, and our results should therefore be regarded as preliminary. Larger populations are required to disentangle the influences of the causes of amputation and the use of prostheses on the cortical reorganization in amputees. Second, as the patients were amputated at different sides, it is inappropriate to perform vertex-based morphological analysis across the whole brain. Third, being a cross-sectional study, we cannot address the intraindividual longitudinal course. Further research with a longitudinal design might help to account more properly for morphometric changes of visual cortex over time. Finally, functional MRI with special behavioral tasks will be helpful to confirm whether the altered brain activity existed in the amputees.

## Conclusion

In this study, high-resolution brain structural MRI was used to investigate the existence and extent of cortical reorganization in subjects with lower limb amputation. We focused on the visual cortex regions and found that amputation of unilateral lower limb may be associated with reduced cortical thickness in the V5/MT+ area. Moreover, we found negative correlation between the time since amputation and cortical thickness of the V5/MT+, indicating that the atrophy of V5/MT+ cortex was progressive. This might be attributed to the degeneration of biological motion perception or tactile motion processing. Though our findings are preliminary and need to be confirmed by future studies, they provide an initial insight into the nature of human adult brain reorganization within cortical cortex after limb injury.

## Author Contributions

XY and JW designed research; GJ, CL, YZ, JW, LL, and XY performed research; TJ, LZ, and AE contributed new analytic tools; GJ, XY, and SR analyzed data; and GJ, XY, and JW wrote the paper. GJ and CL contributed equally to this paper.

## Conflict of Interest Statement

The authors declare that the research was conducted in the absence of any commercial or financial relationships that could be construed as a potential conflict of interest.

## References

[B1] Ajina S. Kennard C. Rees G. Bridge H. (2015). Motion area V5/MT+ response to global motion in the absence of V1 resembles early visual cortex. Brain 138, 164–178. 10.1093/brain/awu328 25433915 PMC4285193

[B2] Becker H. G. Haarmeier T. Tatagiba M. Gharabaghi A. (2013). Electrical stimulation of the human homolog of the medial superior temporal area induces visual motion blindness. J. Neurosci. 33, 18288–18297. 10.1523/JNEUROSCI.0556-13.2013 24227738 PMC6619749

[B3] Binkofski F. Buxbaum L. J. (2013). Two action systems in the human brain. Brain Lang. 127, 222–229. 10.1016/j.bandl.2012.07.007 22889467 PMC4311762

[B4] Binkofski F. Buxbhaum L. J. (2013). Two action systems in the human brain. Brain Lang. 127, 222–229. 10.1016/j.bandl.2012.07.007 22889467 PMC4311762

[B5] Borroni B. Garibotto V. Agosti C. Brambati S. M. Bellelli G. Gasparotti R. (2008). White matter changes in corticobasal degeneration syndrome and correlation with limb apraxia. Arch. Neurol. 65, 796–801. 10.1001/archneur.65.6.796 18541800

[B6] Bridge H. Clare S. Krug K. (2014). Delineating extrastriate visual area MT(V5) using cortical myeloarchitecture. Neuroimage 93(Pt 2), 231–236. 10.1016/j.neuroimage.2013.03.034 23541801

[B7] Bridge H. Thomas O. Jbabdi S. Cowey A. (2008). Changes in connectivity after visual cortical brain damage underlie altered visual function. Brain 131, 1433–1444. 10.1093/brain/awn063 18469021

[B8] Brodie E. E. Whyte A. Waller B. (2003). Increased motor control of a phantom leg in humans results from the visual feedback of a virtual leg. Neurosci. Lett. 341, 167–169. 10.1016/S0304-3940(03)00160-5 12686392

[B9] Chan B. L. Witt R. Charrow A. P. Magee A. Howard R. Pasquina P. F. (2007). Mirror therapy for phantom limb pain. N. Engl. J. Med. 357, 2206–2207. 10.1056/NEJMc071927 18032777

[B10] Cohen L. G. Bandinelli S. Topka H. R. Fuhr P. Roth B. J. Hallett M. (1991). Topographic maps of human motor cortex in normal and pathological conditions: mirror movements, amputations and spinal cord injuries. Electroencephalogr. Clin. Neurophysiol. Suppl. 43, 36–50. 1773774

[B11] Draganski B. Gaser C. Busch V. Schuierer G. Bogdahn U. May A. (2004). Neuroplasticity: changes in grey matter induced by training. Nature 427, 311–312. 10.1038/427311a 14737157

[B12] Draganski B. Moser T. Lummel N. Ganssbauer S. Bogdahn U. Haas F. (2006). Decrease of thalamic gray matter following limb amputation. Neuroimage 31, 951–957. 10.1016/j.neuroimage.2006.01.018 16520065

[B13] Fischl B. Dale A. M. (2000). Measuring the thickness of the human cerebral cortex from magnetic resonance images. Proc. Natl. Acad. Sci. U. S. A. 97, 11050–11055. 10.1073/pnas.200033797 10984517 PMC27146

[B14] Fischl B. Rajendran N. Busa E. Augustinack J. Hinds O. Yeo B. T. (2008). Cortical folding patterns and predicting cytoarchitecture. Cereb. Cortex 18, 1973–1980. 10.1093/cercor/bhm225 18079129 PMC2474454

[B15] Fischl B. Salat D. H. Van Der Kouwe A. J. Makris N. Segonne F. Quinn B. T. (2004). Sequence-independent segmentation of magnetic resonance images. Neuroimage 23(Suppl. 1), S69–S84. 10.1016/j.neuroimage.2004.07.016 15501102

[B16] Fischl B. Sereno M. I. Dale A. M. (1999). Cortical surface-based analysis. II: inflation, flattening, and a surface-based coordinate system. Neuroimage 9, 195–207. 10.1006/nimg.1998.0396 9931269

[B17] Flor H. Denke C. Schaefer M. Grusser S. (2001). Effect of sensory discrimination training on cortical reorganisation and phantom limb pain. Lancet 357, 1763–1764. 10.1016/S0140-6736(00)04890-X 11403816

[B18] Gerber P. Schlaffke L. Heba S. Greenlee M. W. Schultz T. Schmidt-Wilcke T. (2014). Juggling revisited – a voxel-based morphometry study with expert jugglers. Neuroimage 95, 320–325. 10.1016/j.neuroimage.2014.04.023 24736178

[B19] Henriksson L. Karvonen J. Salminen-Vaparanta N. Railo H. Vanni S. (2012). Retinotopic maps, spatial tuning, and locations of human visual areas in surface coordinates characterized with multifocal and blocked FMRI designs. PLoS ONE 7:e36859. 10.1371/journal.pone.0036859 22590626 PMC3348898

[B20] Hinds O. P. Rajendran N. Polimeni J. R. Augustinack J. C. Wiggins G. Wald L. L. (2008). Accurate prediction of V1 location from cortical folds in a surface coordinate system. Neuroimage 39, 1585–1599. 10.1016/j.neuroimage.2007.10.033 18055222 PMC2258215

[B21] Houze B. Bradley C. Magnin M. Garcia-Larrea L. (2013). Changes in sensory hand representation and pain thresholds induced by motor cortex stimulation in humans. Cereb. Cortex 23, 2667–2676. 10.1093/cercor/bhs255 22918979

[B22] Kamitani Y. Tong F. (2006). Decoding seen and attended motion directions from activity in the human visual cortex. Curr. Biol. 16, 1096–1102. 10.1016/j.cub.2006.04.003 16753563 PMC1635016

[B23] Kolster H. Peeters R. Orban G. A. (2010). The retinotopic organization of the human middle temporal area MT/V5 and its cortical neighbors. J. Neurosci. 30, 9801–9820. 10.1523/JNEUROSCI.2069-10.2010 20660263 PMC6632824

[B24] Lund I. Lundeberg T. Sandberg L. Budh C. N. Kowalski J. Svensson E. (2005). Lack of interchangeability between visual analogue and verbal rating pain scales: a cross sectional description of pain etiology groups. BMC Med. Res. Methodol. 5:31. 10.1186/1471-2288-5-31 16202149 PMC1274324

[B25] Maciver K. Lloyd D. M. Kelly S. Roberts N. Nurmikko T. (2008). Phantom limb pain, cortical reorganization and the therapeutic effect of mental imagery. Brain 131, 2181–2191. 10.1093/brain/awn124 18567624 PMC2494616

[B26] Makin T. R. Filippini N. Duff E. P. Henderson Slater D. Tracey I. Johansen-Berg H. (2015). Network-level reorganisation of functional connectivity following arm amputation. Neuroimage 114, 217–225. 10.1016/j.neuroimage.2015.02.067 25776216 PMC4461307

[B27] Makin T. R. Wilf M. Schwartz I. Zohary E. (2010). Amputees “neglect” the space near their missing hand. Psychol. Sci. 21, 55–57. 10.1177/0956797609354739 20424023

[B28] Malikovic A. Amunts K. Schleicher A. Mohlberg H. Eickhoff S. B. Wilms M. (2007). Cytoarchitectonic analysis of the human extrastriate cortex in the region of V5/MT+: a probabilistic, stereotaxic map of area hOc5. Cereb. Cortex 17, 562–574. 10.1093/cercor/bhj181 16603710

[B29] Maunsell J. H. Van Essen D. C. (1983). Functional properties of neurons in middle temporal visual area of the macaque monkey. I. Selectivity for stimulus direction, speed, and orientation. J. Neurophysiol. 49, 1127–1147. 6864242 10.1152/jn.1983.49.5.1127

[B30] Moseley G. L. (2005). Is successful rehabilitation of complex regional pain syndrome due to sustained attention to the affected limb? A randomised clinical trial. Pain 114, 54–61. 10.1016/j.pain.2004.11.024 15733631

[B31] Ninomiya T. Sawamura H. Inoue K. Takada M. (2011). Differential architecture of multisynaptic geniculo-cortical pathways to V4 and MT. Cereb. Cortex 21, 2797–2808. 10.1093/cercor/bhr078 21515714

[B32] Oldfield R. C. (1971). The assessment and analysis of handedness: the Edinburgh inventory. Neuropsychologia 9, 97–113. 10.1016/0028-3932(71)90067-4 5146491

[B33] Palermo L. Di Vita A. Piccardi L. Traballesi M. Guariglia C. (2014). Bottom-up and top-down processes in body representation: a study of brain-damaged and amputee patients. Neuropsychology 28, 772–781. 10.1037/neu0000086 24799290

[B34] Peelen M. V. Downing P. E. (2005). Within-subject reproducibility of category-specific visual activation with functional MRI. Hum. Brain Mapp. 25, 402–408. 10.1002/hbm.20116 15852382 PMC6871698

[B35] Pitzalis S. Galletti C. Huang R. S. Patria F. Committeri G. Galati G. (2006). Wide-field retinotopy defines human cortical visual area v6. J. Neurosci. 26, 7962–7973. 10.1523/JNEUROSCI.0178-06.2006 16870741 PMC6674231

[B36] Pitzalis S. Sereno M. I. Committeri G. Fattori P. Galati G. Tosoni A. (2013). The human homologue of macaque area V6A. Neuroimage 82, 517–530. 10.1016/j.neuroimage.2013.06.026 23770406 PMC3760586

[B37] Preissler S. Feiler J. Dietrich C. Hofmann G. O. Miltner W. H. Weiss T. (2013). Gray matter changes following limb amputation with high and low intensities of phantom limb pain. Cereb. Cortex 23, 1038–1048. 10.1093/cercor/bhs063 22510531

[B38] Rizzolatti G. Matelli M. (2003). Two different streams form the dorsal visual system: anatomy and functions. Exp. Brain Res. 153, 146–157. 10.1007/s00221-003-1588-0 14610633

[B39] Saur D. Kreher B. W. Schnell S. Kummerer D. Kellmeyer P. Vry M. S. (2008). Ventral and dorsal pathways for language. Proc. Natl. Acad. Sci. U. S. A. 105, 18035–18040. 10.1073/pnas.0805234105 19004769 PMC2584675

[B40] Scholz J. Klein M. C. Behrens T. E. Johansen-Berg H. (2009). Training induces changes in white-matter architecture. Nat. Neurosci. 12, 1370–1371. 10.1038/nn.2412 19820707 PMC2770457

[B41] Schwenkreis P. Witscher K. Janssen F. Pleger B. Dertwinkel R. Zenz M. (2001). Assessment of reorganization in the sensorimotor cortex after upper limb amputation. Clin. Neurophysiol. 112, 627–635. 10.1016/S1388-2457(01)00486-2 11275535

[B42] Segonne F. Dale A. M. Busa E. Glessner M. Salat D. Hahn H. K. (2004). A hybrid approach to the skull stripping problem in MRI. Neuroimage 22, 1060–1075. 10.1016/j.neuroimage.2004.03.032 15219578

[B43] Serences J. T. Boynton G. M. (2007). Feature-based attentional modulations in the absence of direct visual stimulation. Neuron 55, 301–312. 10.1016/j.neuron.2007.06.015 17640530

[B44] Stephan K. E. Marshall J. C. Penny W. D. Friston K. J. Fink G. R. (2007). Interhemispheric integration of visual processing during task-driven lateralization. J. Neurosci. 27, 3512–3522. 10.1523/JNEUROSCI.4766-06.2007 17392467 PMC2636903

[B45] Takeuchi H. Taki Y. Hashizume H. Sassa Y. Nagase T. Nouchi R. (2011). Effects of training of processing speed on neural systems. J. Neurosci. 31, 12139–12148. 10.1523/JNEUROSCI.2948-11.2011 21865456 PMC6623215

[B46] Van Kemenade B. M. Seymour K. Wacker E. Spitzer B. Blankenburg F. Sterzer P. (2014). Tactile and visual motion direction processing in hMT+/V5. Neuroimage 84, 420–427. 10.1016/j.neuroimage.2013.09.004 24036354

[B47] Wandell B. A. Dumoulin S. O. Brewer A. A. (2007). Visual field maps in human cortex. Neuron 56, 366–383. 10.1016/j.neuron.2007.10.012 17964252

[B48] Weiner K. S. Grill-Spector K. (2013). Neural representations of faces and limbs neighbor in human high-level visual cortex: evidence for a new organization principle. Psychol. Res. 77, 74–97. 10.1007/s00426-011-0392-x 22139022 PMC3535411

[B49] Wilf M. Holmes N. P. Schwartz I. Makin T. R. (2013). Dissociating between object affordances and spatial compatibility effects using early response components. Front. Psychol. 4:591. 10.3389/fpsyg.2013.00591 24027552 PMC3761160

[B50] Xie H. Kane J. T. Dennis M. J. Mooney R. D. Bauer W. R. Wang X. (2013). Case series evidence for changed interhemispheric relationships in cortical structure in some amputees. J. Clin. Neurosci. 20, 523–526. 10.1016/j.jocn.2012.03.043 23313520

[B51] Yu L. Yin X. Dai C. Liang M. Wei L. Li C. (2014). Morphologic changes in the anterior and posterior subregions of V1 and V2 and the V5/MT+ in patients with primary open-angle glaucoma. Brain Res. 1588, 135–143. 10.1016/j.brainres.2014.09.005 25199592

[B52] Zachariou V. Klatzky R. Behrmann M. (2014). Ventral and dorsal visual stream contributions to the perception of object shape and object location. J. Cogn. Neurosci. 26, 189–209. 10.1162/jocn_a_00475 24001005 PMC5728375

[B53] Zhang M. Y. Katzman R. Salmon D. Jin H. Cai G. J. Wang Z. Y. (1990). The prevalence of dementia and Alzheimer’s disease in Shanghai, China: impact of age, gender, and education. Ann. Neurol. 27, 428–437. 10.1002/ana.410270412 2353798

